# Development of an Enhanced Risk Assessment Model for Human–Robot Collaboration and its Application

**DOI:** 10.1016/j.shaw.2024.12.002

**Published:** 2024-12-26

**Authors:** Kangdon Lee, Jae-Yong Lim

**Affiliations:** 1 SHE DX Division, SK Inc., 41, Cheonggeycheon-ro, Jongro-gu, Seoul, 03188, Republic of Korea; 2 Department of Safety Engineering, Seoul National University of Science and Technology, 232, Gongneung-ro, Nowon-gu, Seoul, 01811, Republic of Korea

**Keywords:** Collaborative robot, Human–robot collaboration, Psychosocial risk, Risk assessment model, Robot safety

## Abstract

**Background:**

Collaborative robots have emerged as a solution for complex and precision-driven tasks. However, there is no comprehensive risk assessment model for collaborative robotic work in the Republic of Korea. Thus, we aim to develop a risk assessment model tailored to the unique characteristics of human-robot collaboration.

**Methods:**

In this study, we examine the risk assessment factors in three key fields: worker, robotic systems, and work environment. The items adopted in the existing models of HFACS, USUS, and human–robot trust were initially selected to extract items for the risk assessment model. The analytic hierarchy process methodology was applied to refine and prioritize the evaluation items. Score scales were constructed for the six checklists. A five-level score ranging from one to five was given to each question in the checklist, and the average score was used to quantitatively evaluate each area.

**Results:**

The six evaluation checklists coalesced to constitute a comprehensive risk assessment model was adjusted for collaborative robot operations. Implementing the proposed model in six robot-operated workplaces yielded consistent results. Companies involved in previous robot-related accidents exhibited deficiencies in these risks than accident-free robot workplaces. Thus, comprehensive risk assessments encompassing the necessary factors are crucial to prevent accidents in robot-related work environments.

**Conclusion:**

The proposed risk assessment model can offer a robust foundation for guiding the future development of diverse risk assessment models for collaborative robot environments and facilitate the safe coexistence of humans and robots in the Industry 4.0 era.

## 1 Introduction

Owing to the different natures of collaborative robot operations in terms of work processes and the perception of risks, it is necessary to evaluate the risks associated with the characteristics of collaborative tasks, such as worker traits, psychology, mutual cooperation, and workspace sharing [1]. Furthermore, shared workspaces and unstructured work environments complicate the risk assessment of collaborative robot operations [2]. However, practical research on this topic is limited [3].

There have been some previous studies to note [[4], [5], [6], [7], [8], [9], [10], [11]]. Lee et al. [4] and Schaefer [5] emphasized that collaborative robot risk assessments should be conducted with sufficient consideration of potential risks in all predictable areas: work organization, collaboration, relationships, work environment, and systems. In a study [6], it was addressed that the risk assessment in collaborative robot operations should have all the predictable risk factors including non-physical hazards like workers' roles, emotional interactions, and organizational communications in robot-operating environments, etc. Through a survey research [7], it was argued that risks in a broader sense (worker interdependencies, organizational reliability, and safety communication, etc.) are as important as physical hazards. However, the previous studies just addressed their importance without including non-physical factors in risk assessments.

The following papers [[8], [9], [10], [11]] began to incorporate non-physical factors into their risk assessments. Sadrfaridpour and Wang [8] and Chemweno et al. [9] tried to consider non-physical risk factors in their risk assessments of collaborative robot operations. However, worker traits and psychosocial aspects were not incorporated in their studies. Some researchers evaluated only limited facets of worker fatigue accumulation, visibility, and transparency of robot operations [10,11]. However, their work did not extensively consider the overall aspects which cannot be neglected in the risk assessment of collaborative robot operations.

In this regard, we present a comprehensive risk assessment model tailored to collaborate robot operations and demonstrate its practical applications. Our proposal on the risk assessment model starts from the recognition that the previous studies does not fully consider non-physical hazards such as workers traits, psychology and organizational communication, etc. The procedure of risk assessment model development is briefly explained as follows. Existing risk assessment items and evaluation methodologies were reviewed from the perspective of worker psychology, robotic systems, and work environment. Candidate items were extracted from the review, and the analytic hierarchy process (AHP) was employed to rank the evaluation items based on the relevance of robot safety, followed by categorizing the evaluation items into nine evaluation factors. Subsequently, six evaluation checklists for the nine factors were developed by referencing existing scale models and customizing the questions to suit collaborative robot operations. The checklists and score scales were integrated, which is the proposed risk assessment model, named as the enhanced human–robot collaboration risk assessment (eHRC-RA) model. eHRC-RA was applied to six robot-using workplaces to validate the evaluation model.

## 2 Materials and methods

### 2.1 Evaluation items

The risk assessment items associated with worker, robotic systems, and work environment were searched using reference models [[12], [13], [14], [15]]. Three representative models suitable for describing these three aspects include: the HFACS model [12], human–robot trust (HRT) model [13,14], and USUS model [15].

**Fig. 1:**
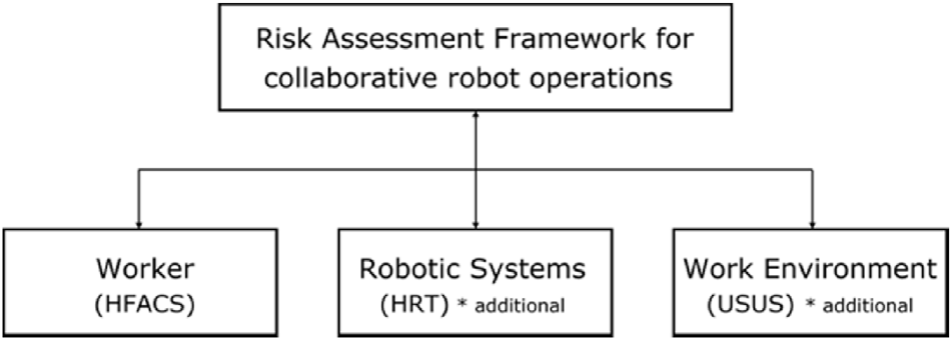
Conceptual diagram of robot collaboration risk assessment model.

The HFACS model is a widely adopted evaluation tool for identifying the root causes of accidents [16] from the perspective of human error. On the other hand, the HRT model assessed the coordination between the capabilities of a robot and human-robot reliability. The USUS model evaluates human-robot environments in the context of collaborative robots. After the review, the group of items classified by **Worker, Robotic system, and Work environment** is called the ‘field’ in this study as shown in Fig. 1.

① **Worker** field: The worker field is handled mainly by selected items from the HFACS model. The HFACS model considers the causes of accidents in the workplace by identifying the underlying factors that contribute to unsafe behavior. Seven items are chosen while the other 13 items were discarded because they were similar to the items selected from HRT and USUS. Instead of the term **Human** used in HFACS and HRT, the term **Worker** will be maintained for the remainder of the article for consistency of terminology.
② **Robotic Systems** field: After reviewing the HRT model, we found that all the items in the HRT model are related to **Robotic Systems** field. Among the 32 items, 29 evaluation items were selected and three items were discarded because they were duplicated with the other two models.
③ **Work Environment** field: A significant proportion of items in the USUS model pertain to the **Work Environment** field. From the 22 items of the USUS model, nine items were selected for evaluation items in the **Work Environment** field. Although the other items are related to **Robotic Systems** field, those are duplicated with the items selected from the HRT model.
④ Evaluation items for each of the three evaluation fields

Overall, 45 evaluation items covering the three fields were maintained to construct the risk assessment model of collaborative robot operations. These included 7 items from the HFACS model, 29 from HRT, and 9 from USUS as shown in Appendix A.

The items within a field were reclassified using evaluation factors, referencing the categorizations of the three existing models. Three evaluation factors in each field reflected the risk aspects of the three fields as a whole. For instance, the **Worker** field has three factors: *personality* (characteristics of workers), *ability* (performance of workers with characteristics), and *behavior* (results of workers performance).

The final evaluation items are listed in Appendix A. The source column indicates the existing model from which each item is extracted, and the priority indicates the most appropriate model if an item exists in more than two models.

### 2.2 Rank items by relevance of robot safety

After selecting the 45 evaluation items, a hierarchical decision-making methodology (AHP) was executed to evaluate the relative importance of the items [17]. A specialist group comprising 21 subject matter experts from manufacturers, users, consulting organizations, and academia was formed. A panel of 13 experts was chosen by utilizing the staticized group research method [18] and Hallowell's point system guideline [19].

The AHP analysis was designed to rank the 45 evaluation items from the perspective of “relevance to robot-related accidents prevention”. Experts were asked to rank 45 evaluation items to prevent worker accidents in collaborative robot work via an online analysis program ‘*I Make It’*.

**Fig. 2:**
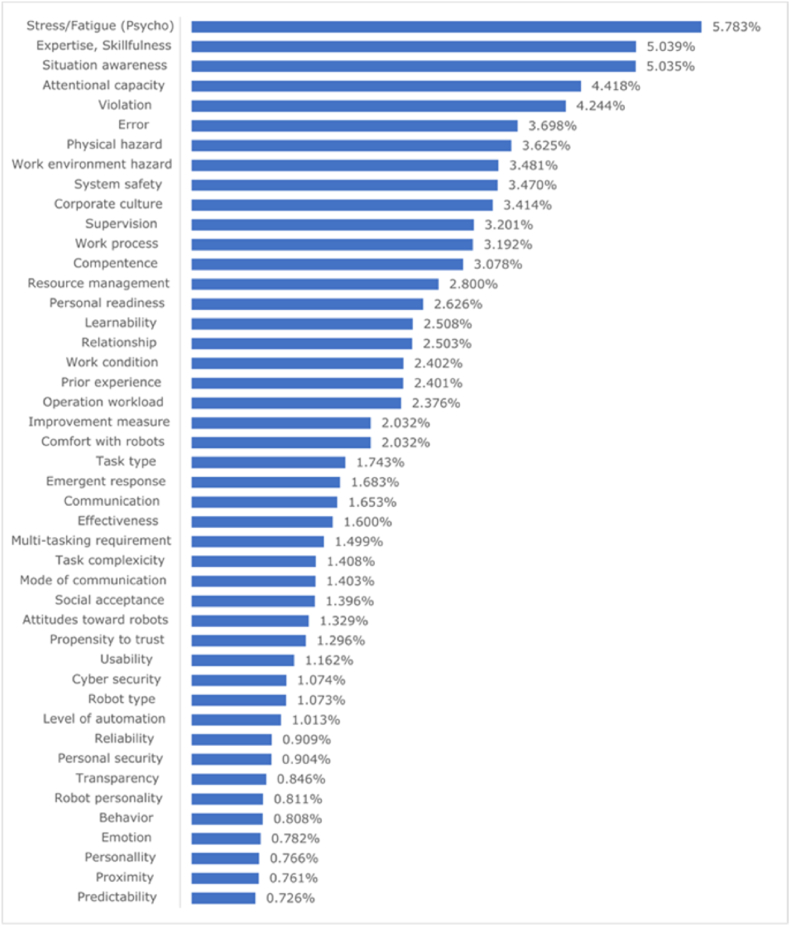
Relative importance ranking results for risk assessment items (AHP analysis).

Fig. 2 displays the AHP results obtained from the program. Experts in the robotics industry consider worker-related risk factors more important than **Robotic Systems** and **Work Environment** fields. Physical and work environmental risks were ranked lower than expected, although they have been emphasized more in conventional risk assessment techniques, such as 4M. Interestingly, the physical risks in **Robotic Systems** field are considered to be less important than risks in **Worker** and those in **Work Environment** fields, which are non-physical risks that have not been considered thus far.

To prioritize the high and low rankings of the evaluation items, the cumulative percentage of items within each factor was used to rank the factors. The top 15 items listed in Appendix B were associated with the following seven factors: *Behavior, Ability, Job/Task, System, Collaboration, Organization, and Personality*. The evaluation items in the two missing factors (*Feature and Capability*) were not closely related to the unconsidered risks in robot safety compared with the items in the other seven factors.

From the AHP analysis, the most important evaluation field for preventing robot accidents was **Worker** (44.9%), followed by **Work Environment** (33.8%), and **Robotic Systems** (21.3%), which is consistent with the Pareto diagram shown in Fig. 2. Overall, the evaluation factors of **Worker** field are the most important as shown in Table 1 and must be identified and resolved in risk assessments of collaborative robot operations.

**Table 1:** Rearrangement of the 45 evaluation items in order of importance

Fields	Factors	Evaluation items		Importance		Source
1. Worker (44.9%)	Personality (6.9%)	Personal readiness	2.62%	15	7th	HRT
		Attitude toward robot	1.32%	31		HRT
		Propensity to trust	1.29%	32		HRT
		Emotion	0.78%	42		USUS
		Trait/disposition	0.76%	43		HRT (Priority)
	Ability (17.9%)	Expertise/amount of training	5.03%	2	2nd	HRT
		Situation awareness	5.03%	3		HRT
		Competence	3.07%	13		HRT
		Prior experience	2.40%	19		HRT
		Operator workload	2.37%	20		HRT
	Behavior (20.1%)	Stress/fatigue	5.78%	1	1st	HRT
		Attention capacity/engagement	4.41%	4		HRT
		Violation	4.24%	5		HFACS
		Error	3.69%	6		HFACS
		Comfort with robot	2.03%	21		HRT
2. Robotic systems (21.3%)	Feature (5.5%)	Mode of communication	1.40%	29	8th	HRT
		Usability	1.16%	33		USUS
		Robot type	1.07%	35		HRT(Priority)
		Level of automation	1.01%	36		HRT
		Robot personality	0.81%	40		HRT
	Capability (4.1%)	Reliability	0.90%	37	9th	HRT(Priority)
		Transparency	0.84%	39		HRT
		Robot behavior	0.80%	41		HRT
		Proximity/co-location	0.76%	44		HRT
		Predictability	0.72%	45		HRT
	Job/task (11.7%)	Physical hazard	3.62%	7	4th	HRT
		Environment hazard	3.48%	8		HRT
		Task type	1.74%	23		HRT
		Multi-tasking requirement	1.49%	27		HRT
		Task complexity	1.40%	28		HRT
3. Work environment (33.8%)	Organization procedure (13.7%)	Supervision	3.20%	11	3rd	HFACS
		Process/procedure	3.19%	12		HFACS
		Resource management	2.80%	14		HFACS
		Working condition	2.40%	18		USUS
		Correction	2.03%	21		HFACS
	Collaboration communication (10.6%)	Culture	3.41%	10	5th	HRT(Priority)
		Relationship	2.50%	17		HRT(Priority)
		Emergency response	1.68%	24		HFACS
		Communication	1.65%	25		HRT(Priority)
		Social acceptance	0.64%	30		USUS(Priority)
	System (9.5%)	System safety	3.47%	9	6th	USUS(Priority)
		Learnability	2.50%	16		USUS
		Effectiveness	1.60%	26		USUS
		Cyber security	1.07%	34		USUS
		Personal security	0.90%	38		USUS

HRT, human–robot trust.

### 2.3 Development of evaluation scales

The 9 evaluation factors encompassing all 45 items required quantitative evaluation using the corresponding evaluation scale, which was in the form of a checklist. Nine evaluation factors were assessed using six evaluation checklists customized based on the reference scale models (rightmost column in Table 2). The fourth evaluation factor of *Job/Task* is not associated with any evaluation checklist because the risks in the evaluation factor, whose primary focuses are physical risks, have been assessed using conventional methods, such as 4M and the risk graph method [20].

**Table 2:** Classification of evaluation ranking factors and evaluation checklists

Evaluation fields	Evaluation factors (sum percentage of importance ratio)		Evaluation checklist
1. Worker (W) (44.9%)	Personality (6.9%)	7th Factor	Personality
	Ability (17.9%)	2nd Factor	Psychology
	Behavior (20.1%)	1st Factor	
2. Robotic (R) systems (21.3%)	Feature (5.5%)	8th Factor	Robot trust
	Capability (4.1%)	9th Factor	
	Job/task (11.7%)	4th Factor	N/A (exclusion)
3. Work (E) environment (33.8%)	Organization, procedure (13.7%)	3rd Factor	Organization and Procedures
	Collaboration, communication (10.6%)	5th Factor	Collaboration and Communication
	System (9.5%)	6th Factor	Cybersecurity

Additionally, the two checklists of psychology and robot trust includes two distinctive factors, *Ability* and *Behavior*, *Feature* and *Capability*, respectively. Therefore, the six checklists and a conventional risk assessment method can manage all the factors related to collaborative robot operations.

Each evaluation checklist consisted of 20 questions, and overall 120 questions. In this study, the six evaluation checklists were named personality, psychology, robot trust, organization and procedures, collaboration and communication, and cybersecurity as listed in Table 2. The risks neglected thus far can be quantitatively assessed using six evaluation checklists. However, robot-related jobs and task (4th factor) were excluded from the evaluation as mentioned in Section 2.3.

### 2.4 Scoring system of evaluation scale

#### 2.4.1 Personality checklist

The original version of the mini-IPIP was adopted to evaluate the personality factors of *Personality* associated with the personality checklist. As the mini-IPIP, consisting of 20 questions, is the best for evaluating personality traits in various research fields [21], the appraisal of worker personality, attitude, conscientiousness, and other traits is expected to be effective in the context of collaborative robot operations. Application of the personality checklist to the workforce can gauge employee personality and disposition by completing the checklist on a five-point scale. The details of each checklist group are provided in Appendix C.

All questions in the personality checklist should be answered on a five-level scale. However, some question numbers (5–8, 12–16, 18, and 20) in the personality checklist are reverse-scored questions. The scores for the four questions in each personality group were then averaged and later used as a basis for the corresponding checklist group. Consequently, the personality checklist can assess the evaluation factors of *Personality* in the field of **Worker**.

#### 2.4.2 Psychological checklist

The Dundee Stress State Questionnaire (DSSQ) scale [22] was chosen as the fundamental framework that explains stress, fatigue, and cognition. DSSQ, which has been validated in the psychology research field [23], has two types of scales: a full version (96 questions) and a short version, (SSSQ: 16 questions).

There is a complication in using the full version of DSSQ because of too many questions. Thus, we customized a checklist of 20 questions by adding four questions to the 16 questions of SSSQ, which are pertinent to psychological risks about collaborative robot operations. A checklist named psychology is provided in Appendix D. The 20 questions were related to the four checklist groups as shown in Appendix C, so that there were five questions in each checklist group. All questions were scored individually on a five-point scale as explained in Section 2.4.1. Questions 1, 2, 5–7, and 14–16 are reverse-scored questions.

Upon the completion of the psychology checklist, the scores of questions in each checklist group are averaged. A checklist group with a high average value indicated a good state regarding the checklist group. In contrast, a checklist group of fewer than three warns that there needs to be corresponding company-level work and psychological measures. Consequently, the psychology checklist can assess the evaluation factors of *Ability* and *Behavior* in the field of **Worker**.

#### 2.4.3 Robot trust checklist

The HRT model [24] gauges the trust level of the worker in the transparency, interactivity, and reliability of robotic systems within collaborative environments. To construct our checklist, the HRT scale was selected as the framework owing to its guaranteed performance. The primary attribute is highlighted not only in the ability to calibrate the levels of trust but also in the system capabilities of facilitating the appropriate usage of robotic systems. The HRT scale has two versions: a short version (14 questions) and a full version (40 questions).

The short version does not incorporate critical features, such as ease of use, proximity, and predictability in collaborative robot operations. Thus, six questions from the full version were added to the short version of the HRT [5], resulting in 20 questions for the checklist. As shown in Appendix C, the 20 questions were categorized into five checklist groups with four questions per checklist group. The customized checklist of 20 questions is called the Robot trust checklist (Appendix D).

In the robot trust checklist, each question is scored on a five-point scale and the question numbers of 3, 4, 13, 19, and 20 are reverse-scored. The scores of the four questions within each checklist group were then averaged to obtain the results with respect to the individual checklist group.

As done previously, the average in each checklist group can be interpreted as follows: If there is a particular checklist group with a low average, improvement measures should be implemented to enhance the corresponding features and capabilities of the robot system. Consequently, the robot trust checklist can be used to assess the evaluation factors of *Feature* and *Capability* in the field of **Robotic Systems.**

#### 2.4.4 Organization and procedures checklist

Our checklist for evaluating the factors of *Organization and Procedures* (Table 2) was based on the psychosocial risk guidelines of ISO 45003 [25]. First, 36 assessment items presented as examples of psychosocial risks in clause 6.1.2.1 of ISO 45003 were examined. There are approximately eight organization procedure-related risks among the psychosocial impact factors in the enterprise. Then, 20 out of 36 were selected for constructing the checklist with five checklist groups as shown in Appendix C. The checklist of 20 questions is called the organization and procedures checklist (Appendix D).

The scoring method and interpretation of the results are as explained in previous checklists. All questions in the organization and procedure checklist were scored on a five-point scale and eight questions were reverse-scored. The average scores of the questions in the same checklist groups were then calculated and used as a basis for preventive measures. Consequently, the organization and procedure checklist can assess the evaluation factors of *Organization and Procedures* in the **Work Environment** field.

#### 2.4.5 Collaboration and communication checklist

The checklist for evaluating factors of *Collaboration and Communication* was based on the 32 assessment items in Section 6.1.2.1 of ISO 45003. After the analysis, 20 out of the 32 assessment items were selected, which were closely related to interpersonal relationships, leadership, organizational culture, recognition and rewards, and management and supervision. Thus, a checklist consisting of 20 questions called the Collaboration and Communication checklist was formed (Appendix D). The 20 questions were categorized as shown in Appendix C.

The scoring method and interpretation of the results were similar to that of the previous checklists. Each question on a five-point scale and ten reverse-scored questions are indicated. The average scores for the four questions in the same checklist group were then obtained.

For a checklist group with an average score lower than 3, appropriate strategies must be employed to bolster the degree of collaboration and communication within the organization. These strategies include improving intra-employee communication, augmenting managerial leadership, fostering a positive safety climate, and providing superior rewards and support. The collaboration and communication checklist can assess the evaluation factors of *Collaboration and Communication* in the **Work Environment.**

#### 2.4.6 Cybersecurity checklist

The checklist for evaluating factors of the *System* was constructed based on the NIST cybersecurity checklist [26]. Inclusive of robot cyber securities in the original 20 questions, a modified version of the checklist called as the cybersecurity checklist was created (Appendix D). The modified questions were divided into four checklist groups as shown in Appendix C.

All 20 questions were scored on a five-point scale. Similarly, the average score of the four questions in the same checklist group played a key role in evaluating the corresponding checklist group. If the average score in a certain checklist group is less than three, appropriate actions should be taken, including strengthening security verification procedures against cyber-attacks, and enhancing regular backup and response protocols for security incidents. Consequently, the cybersecurity checklist can be used to assess the evaluation factors of *System* in the **Work Environment** field.

## 3 Results and discussion

**Fig. 3:**
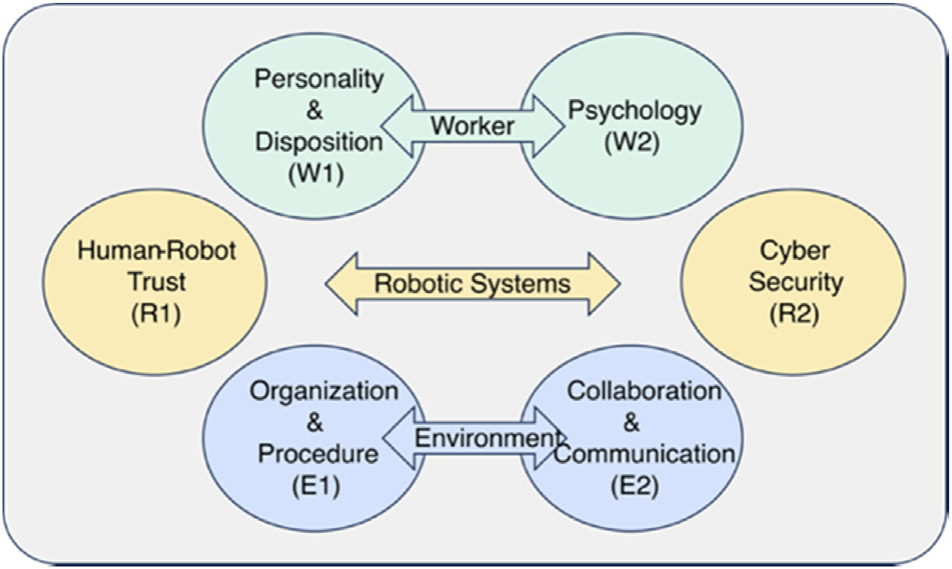
Conceptual diagram of the eHRC-RA model.

Finally, six comprehensive evaluation checklists encompassing 45 evaluation items associated with collaborative robot operations were constructed based on six corresponding evaluation reference models. The eHRC-RA model was developed by integrating six evaluation checklists as shown in Fig. 3.

### 3.1 Validation of eHRC-RA model

To validate the eHRC-RA model, the proposed risk assessment was applied to six workplaces, four enterprises with robot accidents in the past, and two companies without accidents. Interviews were conducted with 3–4 robot-related workers at each business site. Each interviewee was asked 120 questions. The details of the application are provided in another paper.

**Fig. 4:**
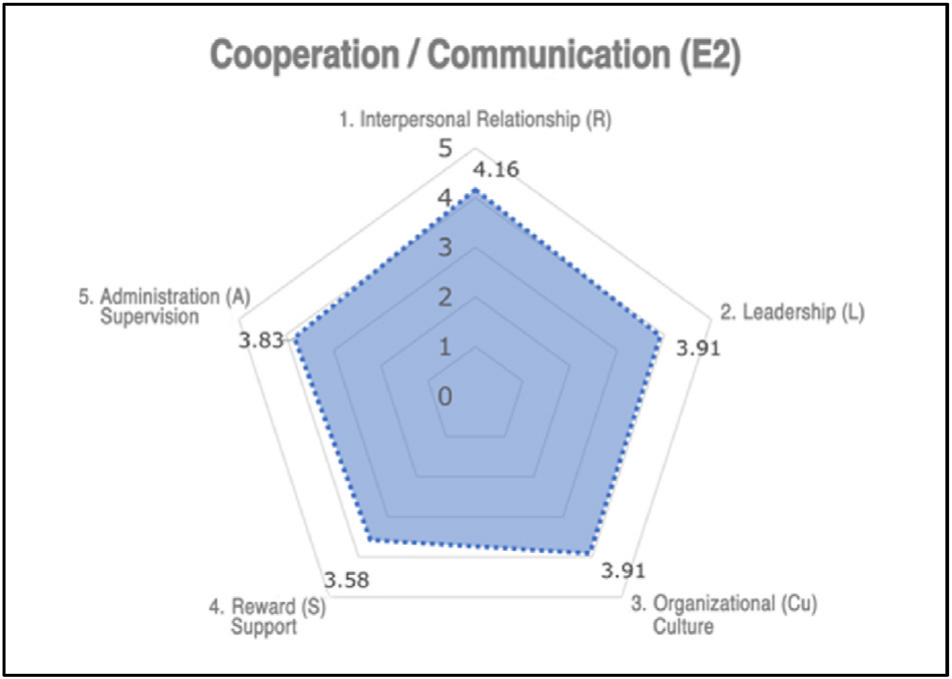
Positive state (no fatality).

**Fig. 5:**
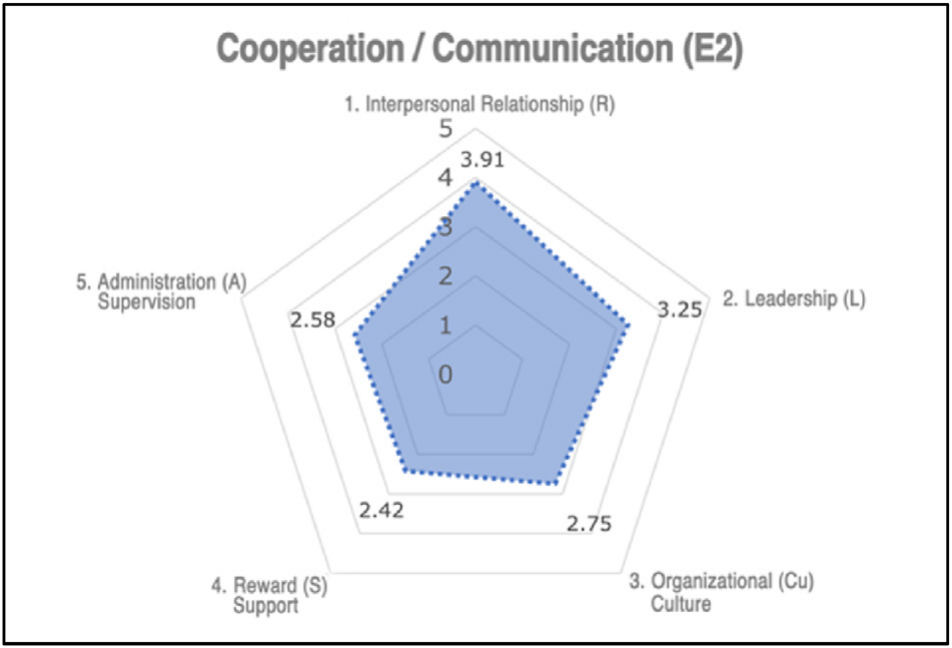
Improper state (one fatality by robot).

The evaluation results analyzed using the SPSS statistical program are visualized in radar graphs shown in Fig. 4, Fig. 5. This leads to the final determination of the current status of each checklist group and the necessity for improvement.

For the two accident-free workplaces, there were no serious problems with the checklist groups. Additionally, from the application of the eHRC-RA model to four workplaces where robotic accidents occurred, several aspects were found to be unsatisfactory: safety and health vulnerabilities in **Worker, Robotic systems, and Work environment** fields. However, these aspects cannot be identified using existing risk assessments.

Companies can utilize the assessment results as a basis for decision-making to prevent the recurrence of similar accidents associated with robotic systems. Our proposed model is expected to facilitate the implementation of fundamental improvement measures for the vulnerabilities of robot-related work. Moreover, eHRC-RA serves as an alternative evaluation model that supplements existing evaluation results by analyzing risks that cannot be assessed using conventional methods such as 4M.

### 3.2 Discussion

We proposed the eHRC-RA model as a risk assessment technique that includes not only existing visible physical risks but also workers and environmental risks. Our contribution is developing a risk assessment technique capable of assessing worker disposition and psychological state, worker-robot reliability and cybersecurity, organization and procedures, and collaboration and communication. These risks are strongly related to the root causes of robot accidents, and the proposed eHRC-RA model aims to prevent accidents during collaborative robot operations.

Using the six evaluation checklists, the eHRC-RA model facilitates the comprehensive assessment and quantification of all risk factors. Despite their significance, the identification of these factors has been neglected in conventional risk assessment methods.

To apply the eHRC-RA technique in the field, six workplaces were selected, including four sites where robot-related accidents had occurred and two sites with no reported accidents. In the actual field evaluation, the robot work processes and operational conditions were verified by the supervisor through on-site 30 minutes meeting. Subsequently, a 15-minute instruction was followed for 3-4 workers who were supposed to participate in the eHRC-RA evaluation, in which the purpose of the eHRC-RA technique and the evaluation method are outlined. Then, the eHRC-RA checklists (6 pages) were given to them. It took about 15 minutes to complete the assessment (10–12 minutes on average).

The application of the six evaluation checklists in companies experiencing robot-related accidents enables identifying deficiencies in non-physical risk factors. These were diagnosed using radar graphs that visualized the strengths and weaknesses of each company. Subsequently, appropriate improvement measures can be implemented to address the indicated deficiencies, bolster overall safety, and mitigate the likelihood of future incidents.

Application of the eHRC-RA model demonstrated that the root cause of robot accidents was a primary causal factor of robot-related worker accidents. These underlying factors, which were not considered in conventional 4M risk assessment, were effectively explained by applying the eHRC-RA model. Our proposed model can provide valuable insights into the multifaceted nature of robot accidents and aid in proper interventions to mitigate future occurrences.

However, application of the eHRC-RA model to other machinery is limited because this model is optimized for collaborative robot operations. Particularly, 35 of the 120 questions were specific to robot-related works. Also, some restrictions of this study exist in the number of companies: the appropriate proportions of experimental and control groups and in the consideration of the experience and expertise of the participants.

To expand the proposed model to other industries, it is necessary to revise the evaluation factors and checklist questions specific to collaborative robotic environments to reflect the characteristics of other industries. Finally, various types of work, such as gender, robot work contracting, rental, and consignment, which do not strictly belong to the three evaluation fields, may influence the safety of collaborative robot operations.

## 4 Conclusion

We propose a comprehensive risk assessment model, eHRC-RA, to assess previously unrecognized risks in collaborative robot environments. The efficacy of the eHRC-RA model supplements the evaluation of non-physical risks that established methods cannot consider as a critical role of non-physical risk assessment in robot-related accident prevention.

First, a novel risk assessment model is presented that encompasses three key evaluation fields: **Worker, Robotic Systems,** and **Work Environment**. The model is based on international standards and includes evaluation factors that can assess worker traits, psychology, organizational communication, and work environment. This risk assessment model offers a decisive solution to previous concerns that current risk assessment techniques cannot properly identify all risks, including the root causes of robot-related accidents in the workplace.

Second, we developed a risk assessment model, eHRC-RA, that includes non-physical evaluation fields related to robot collaboration. This model was effective in identifying the root causes of robot-related accidents by applying eHRC-RA to six companies in which robot accidents occurred over the past three years.

Third, the AHP results showed that the field of **Worker** is the most important evaluation field in mitigating robot-related accidents, compared with the fields of **Robotic Systems** and that of **Work Environment**. The significance of **Worker** amounted to 44.9% of the total percentage, whereas those of **Work Environment** and **Robotic Systems** was 33.8% and 21.3%, respectively.

Overall, identifying physical risks using established techniques and the inclusion of non-physical factors will enhance accident prevention measures in collaborative robot operations. By identifying the root causes of robot-related accidents, the risk assessment of non-physical risks offers a substantial contribution toward improving the safety outcomes of collaborative work environments.

## CRediT authorship contribution statement

**Kangdon Lee:** Writing – original draft. **Jae-Yong Lim:** Writing – review & editing, Supervision.

## Conflicts of interest

The authors declare no conflict of interest.
