# Supplementary material for: Development of an Enhanced Risk Assessment Model for Human–Robot Collaboration and its Application
Source: Saf Health Work. 2024 Dec 26;16(1):83–9. doi: 10.1016/j.shaw.2024.12.002 (PMC11959558; doi:10.1016/j.shaw.2024.12.002)
Supplement: Multimedia component 1 [file mmc1.docx]

**Appendix A. Items of risk assessment for collaborative robot work**

| Fields | Factors | Evaluation items | Source |
| --- | --- | --- | --- |
| 1. Worker | Personality | Trait, disposition | HRT (Priority) |
|  |  | Attitudes toward robot | HRT |
|  |  | Propensity to trust | HRT |
|  |  | Emotion | USUS |
|  |  | Personal readiness | HRT |
|  | Ability | Expertise, amount of training | HRT |
|  |  | Competence | HRT |
|  |  | Prior experience | HRT |
|  |  | Operator workload | HRT |
|  |  | Situation awareness | HRT |
|  | Behavior | Attention capacity, engagement | HRT |
|  |  | Stress, fatigue | HRT |
|  |  | Error | HFACS |
|  |  | Violation | HFACS |
|  |  | Comfort with robot | HRT |
| 2. Robotic  systems | Feature | Level of automation | HRT |
|  |  | Robot type | HRT (Priority) |
|  |  | Robot personality | HRT |
|  |  | Mode of communication | HRT |
|  |  | Usability | USUS |
|  | Capability | Robot behavior | HRT |
|  |  | Reliability | HRT (Priority) |
|  |  | Predictability | HRT |
|  |  | Proximity, co-location | HRT |
|  |  | Transparency | HRT |
|  | Task | Task type | HRT |
|  |  | Task complexity | HRT |
|  |  | Multi-tasking requirement | HRT |
|  |  | Physical hazard | HRT |
|  |  | Environment hazard | HRT |
| 3. Work  environment | Organization  procedure | Resource management | HFACS |
|  |  | Working condition | USUS |
|  |  | Process, procedure | HFACS |
|  |  | Supervision | HFACS |
|  |  | Correction | HFACS |
|  | Collaboration  communication | Culture | HRT (Priority) |
|  |  | Social acceptance | USUS (Priority) |
|  |  | Communication | HRT (Priority) |
|  |  | Relationship | HRT (Priority) |
|  |  | Emergency response | HFACS |
|  | System | System safety | USUS (Priority) |
|  |  | Learnability | USUS |
|  |  | Effectiveness | USUS |
|  |  | Cyber security | USUS |
|  |  | Personal security | USUS |

**Appendix B. Top 15 important evaluation items among the 45 evaluation items**

| Fields | Evaluation items | (Factor) | Ranking |
| --- | --- | --- | --- |
| Worker (W) | Stress/fatigue | (Behavior) | 1 |
|  | Expertise/amount of training | (Ability) | 2 |
|  | Situation awareness | (Behavior) | 3 |
|  | Attention capacity/engagement | (Behavior) | 4 |
|  | Error | (Behavior) | 5 |
|  | Violation | (Behavior) | 6 |
| Robot (R) | Physical hazard | (Job/Task) | 7 |
|  | Environment hazard | (Job/Task) | 8 |
| Environment (E) | System safety | (System) | 9 |
|  | Corporate culture | (Collaboration) | 10 |
|  | Supervision | (Organization) | 11 |
|  | Process/procedure | (Organization) | 12 |
| Worker (W) | Competence | (Ability) | 13 |
| Environment (E) | Resource management | (Organization) | 14 |
| Worker (W) | Personal readiness | (Personality) | 15 |

**Appendix C. Checklist groups of 6 evaluation checklist**

| Checklist Group | | Meaning |
| --- | --- | --- |
| 1. Personality  checklist | ① Extroversion | A tendency to speak up and enjoy interacting with colleagues |
|  | ② Agreeableness | A penchant to talk about and empathize with surrounding tasks and coworker situations |
|  | ③ Imagination | Enjoys making suggestions, discussing ideas, and trying new things |
|  | ④ Conscientiousness | Tendency to get things done quickly and thoroughly |
|  | ⑤ Neuroticism | Nervous, demanding, and self-centered. |
| 2. Psychological  checklist | ① Mood state  (stress, fatigue) | Assess highs and lows of mood, fatigue, and stress, including complaints, depression, worry, and anxiety. |
|  | ② Motivation  (attitude) | Evaluate the internal and external motivation of each individual. |
|  | ③ Cognition  (concentration) | Assessment of individual cognitive styles, such as attention, focus, and self-esteem. |
|  | ④ Cognition  (thoughts) | Assessment of personalized cognitive content on and off task |
| 3. Robot trust  checklist | ① Reliability | The degree to which a robotic system is reliable, including consistency of behavior, the occurrence of errors, and accurate performance. |
|  | ② Transparency | Transparency in the performance of tasks, such as successfully performing functions, achieving goals, and fulfilling orders. |
|  | ③ Interaction | Reliance on workers to provide task feedback, relevant information, communication, etc. |
|  | ④ Automation | Increase automation for proximity, teammate recognition, detection, control, and actuation. |
|  | ⑤ Predictability | Predictability of breakdowns, maintenance needs, etc. |
| 4. System  checklist | ① Risk identification | Procedures and devices to verify and identify the risk of cybersecurity incidents |
|  | ② Risk protection | Procedures and devices to protect employees, facilities, and information from security incidents |
|  | ③ Risk detection &  response | Measures and devices to quickly detect and respond to security incident risks |
|  | ④ Risk recovery | Regular backup and post-incident response procedures in case of security issues. |
| 4. Organization and Procedures  checklist | ① Less role expectations | Low expectations, such as job role delineation, existence and clarity of standard procedures, and watchful for others. |
|  | ② Less job demands  (less business needs) | Low demands, including deadline pressure, sensitive tasks and coworkers, and multiple workers simultaneously |
|  | ③ Organizational  change | Organizational support and change management, including shifts, staffing, communicating changes, and working alone. |
|  | ④ Less workload | Less overtime, pressure to get things done, deadlines, pacing, etc. |
|  | ⑤ Flexible  work schedule | Less tight schedules, overtime, uniformity of work, fragmentation, etc. |
| 5. Collaboration and Communication checklist | ① Interpersonal  relationships | Positive relationships, including communication between employees, amicable relationships, and no harassment/bullying. |
|  | ② Leadership | Operationalize safety and health leadership, including visioning, goal setting, decision making, and executive communication. |
|  | ③ Organizational  culture | Create a culture of safety in your organization, align goals, support problem solving, and create a safe environment |
|  | ④ Recognition,  rewards | Recognition of effort, performance, rewards, and development. |
|  | ⑤ Management,  supervision | Provide oversight and effectiveness, including goal sharing, feedback, encouragement, recognition, etc. |

**Appendix D. Six evaluation checklists of eHRC-RA model**

(1) Personality checklist (mini-IPIP)

* Strongly agree = 5 Agree = 4 Neither agree nor disagree = 3 Disagree = 2 Strongly disagree = 1

| No. | Big Five Factors of Personality  *R: Reverse-scored question | Answer for personality | | | | | Remarks |
| --- | --- | --- | --- | --- | --- | --- | --- |
|  |  | 5 | 4 | 3 | 2 | 1 |  |
| 1 | I am the life of the party. |  |  |  |  |  | Extraversion |
| 2 | I sympathize with others’ feelings. |  |  |  |  |  | Agreeableness |
| 3 | I get chores done right away. |  |  |  |  |  | Conscientiousness |
| 4 | I have a vivid imagination. |  |  |  |  |  | Intellectl/Imagination |
| 5 | I don’t talk a lot. *R |  |  |  |  |  | Extraversion |
| 6 | I am not interested in other people’s problems. *R |  |  |  |  |  | Agreeableness |
| 7 | I often forget to put things back in their proper place. *R |  |  |  |  |  | Conscientiousness |
| 8 | I am not interested in abstract ideas. *R |  |  |  |  |  | Intellect/Imagination |
| 9 | I talk to a lot of different people at parties. |  |  |  |  |  | Extraversion |
| 10 | I feel others’ emotions. |  |  |  |  |  | Agreeableness |
| 11 | I like order. |  |  |  |  |  | Conscientiousness |
| 12 | I have difficulty understanding abstract ideas. *R |  |  |  |  |  | Intellect/Imagination |
| 13 | I keep in the background. *R |  |  |  |  |  | Extraversion |
| 14 | I am not really interested in others. *R |  |  |  |  |  | Agreeableness |
| 15 | I make a mess of things. *R |  |  |  |  |  | Conscientiousness |
| 16 | I don’t have a good imagination. *R |  |  |  |  |  | Intellect/Imagination |
| 17 | I have frequent mood swings. |  |  |  |  |  | Neuroticism |
| 18 | I am relaxed most of the time. *R |  |  |  |  |  | Neuroticism |
| 19 | I get upset easily. |  |  |  |  |  | Neuroticism |
| 20 | I seldom feel blue. *R |  |  |  |  |  | Neuroticism |

(2) Psychology checklist

* Strongly agree = 5 Agree = 4 Neither agree nor disagree = 3 Disagree = 2 Strongly disagree = 1

| No. | Stress state during collaborative robot operations  (Mood, Motivation, Thinking style, Thinking content)  *R: Reverse-scored question | Answer for stress state | | | | | Remarks |
| --- | --- | --- | --- | --- | --- | --- | --- |
|  |  | 5 | 4 | 3 | 2 | 1 |  |
| 1 | I am not being tired and not being grouchy. *R |  |  |  |  |  | Stress, Fatigue |
| 2 | I am being satisfied with work environment. *R |  |  |  |  |  | Stress, Fatigue |
| 3 | I am being anxious, jittery, and depressed. |  |  |  |  |  | Stress, Fatigue |
| 4 | I am being nervous and being alert during robot-related tasks. |  |  |  |  |  | Stress, Fatigue |
| 5 | I am being active and energetic during robot-related tasks. *R |  |  |  |  |  | Stress, Fatigue |
| 6 | I would rather spend the time doing the task on something else. *R |  |  |  |  |  | Motivation |
| 7 | I will become fed up with robot-related tasks. *R |  |  |  |  |  | Motivation |
| 8 | I am committed to attaining my performance goals. |  |  |  |  |  | Motivation |
| 9 | I want to succeed on the task. |  |  |  |  |  | Motivation |
| 10 | I am motivated to do the task. |  |  |  |  |  | Motivation |
| 11 | I am trying (tried) to figure myself out. |  |  |  |  |  | Cognition  (Concentration) |
| 12 | I am reflecting (reflected) about myself. |  |  |  |  |  | Cognition  (Concentration) |
| 13 | I am daydreaming about myself. |  |  |  |  |  | Cognition  (Concentration) |
| 14 | I am worried about what other people think of me. *R |  |  |  |  |  | Cognition  (Concentration) |
| 15 | My attention is directed towards things other than robot-related tasks. *R |  |  |  |  |  | Cognition  (Concentration) |
| 16 | My mind is wandering (wandered) a great deal. *R |  |  |  |  |  | Cognition  (Thoughts) |
| 17 | I think about how others have done on this task. |  |  |  |  |  | Cognition  (Thoughts) |
| 18 | I think about my feeling of being told how I performed. |  |  |  |  |  | Cognition  (Thoughts) |
| 19 | I think about personal worries during robot-related tasks. |  |  |  |  |  | Cognition  (Thoughts) |
| 20 | I think about something that happened recently. |  |  |  |  |  | Cognition  (Thoughts) |

(3) Robot Trust checklist

* A great deal(80~100%) = 5 Quite a bit(60~80%) = 4 Sometimes(40~60%) = 3 Very little(20~40%) = 2 Not at all(0~20%) = 1

| No. | Robot Trust  (What % of the time will robots...)  *R: Reverse-scored question | Answer for Human-Robot Trust | | | | | Remarks  (*: Short HRT) |
| --- | --- | --- | --- | --- | --- | --- | --- |
|  |  | 5 | 4 | 3 | 2 | 1 |  |
| 1 | Most robots act consistently. |  |  |  |  |  | Reliability* |
| 2 | Most robots function successfully. |  |  |  |  |  | Transparency* |
| 3 | Most robots malfunction. *R |  |  |  |  |  | Transparency* |
| 4 | Most robots have errors. *R |  |  |  |  |  | Reliability* |
| 5 | Most robots provide feedback. |  |  |  |  |  | Interaction* |
| 6 | Most robots meet the needs of the mission. |  |  |  |  |  | Transparency* |
| 7 | Most robots provide appropriate information. |  |  |  |  |  | Interaction* |
| 8 | Most robots communicate with people. |  |  |  |  |  | Interaction* |
| 9 | Most robots perform exactly as instructed. |  |  |  |  |  | Reliability* |
| 10 | Most robots follow directions. |  |  |  |  |  | Transparency* |
| 11 | Most robots are dependable. |  |  |  |  |  | Interaction* |
| 12 | Most robots are reliable. |  |  |  |  |  | Reliability* |
| 13 | Most robots are unresponsive. *R |  |  |  |  |  | Predictibility* |
| 14 | Most robots are predictable. |  |  |  |  |  | Predictibility* |
| 15 | Most robots work in close proximity with people. |  |  |  |  |  | Automation |
| 16 | Most robots appear to be conscious (or make sensible decisions). |  |  |  |  |  | Automation |
| 17 | Most robots are considered part of the team. |  |  |  |  |  | Automation |
| 18 | Most robots are autonomous. |  |  |  |  |  | Automation |
| 19 | Most robots require frequent maintenance. *R |  |  |  |  |  | Predictibility |
| 20 | Most robots are easily led astray by unexpected changes in environment or task. *R |  |  |  |  |  | Predictibility |

(4) Cybersecurity checklist

* Strongly agree = 5 Agree = 4 Neither agree nor disagree = 3 Disagree = 2 Strongly disagree = 1

| No. | Cybersecurity for robot-related tasks  *R: Reverse-scored question | Answer for cybersecurity | | | | | Remarks |
| --- | --- | --- | --- | --- | --- | --- | --- |
|  |  | 5 | 4 | 3 | 2 | 1 |  |
| 1 | Robotic systems don’t identify and control who has access to business information. *R |  |  |  |  |  | Risk  identification |
| 2 | Robotic systems conduct background checks for robot operators. |  |  |  |  |  | Risk  identification |
| 3 | Robotic systems don’t require individual user accounts for each employee. *R |  |  |  |  |  | Risk  identification |
| 4 | There are created policies and procedures of cybersecurity for robot-related tasks. |  |  |  |  |  | Risk  identification |
| 5 | Robotic systems limit employees access to data and information on robots. |  |  |  |  |  | Risk  identification |
| 6 | Robotic systems don’t install surge protectors and UPS. *R |  |  |  |  |  | Risk  protection |
| 7 | Robotic systems secure your wireless access point and networks. |  |  |  |  |  | Risk  protection |
| 8 | Robotic systems set up web and email filters and use encryption for sensitive information. |  |  |  |  |  | Risk  protection |
| 9 | My company disposes of robotic systems, old computers, and media with others altogether. *R |  |  |  |  |  | Risk  protection |
| 10 | My company trains its employees about robotic systems and cybersecurity. |  |  |  |  |  | Risk  protection |
| 11 | My company doesn’t install anti-virus, anti-spyware, etc. in robotic systems. *R |  |  |  |  |  | Detect & Respond |
| 12 | Robotic systems update anti-virus, anti-malware programs, etc regularly. |  |  |  |  |  | Detect & Respond |
| 13 | Robotic systems monitor robot-related tasks and maintain networking logs. |  |  |  |  |  | Detect & Respond |
| 14 | Response plan of my company is excuted during or after security incidents. |  |  |  |  |  | Detect & Respond |
| 15 | Personnel of my company know their roles and order of operations after security incidents. *R |  |  |  |  |  | Detect & Respond |
| 16 | My company has a emergency plan for disasters and information security incidents. |  |  |  |  |  | Risk  recovery |
| 17 | Robotic systems make full backups of important business data and robot-related information. |  |  |  |  |  | Risk  recovery |
| 18 | My company doesn’t continue to schedule incremental backups. *R |  |  |  |  |  | Risk  recovery |
| 19 | My company doesn’t consider cybersecurity insurance for the failure of robotic systems . *R |  |  |  |  |  | Risk  recovery |
| 20 | My company has a plan to improve processes, procedures, and technologies for secure robotic systems. |  |  |  |  |  | Risk  recovery |

(5) Organization and Procedures checklist

* Strongly agree = 5 Agree = 4 Neither agree nor disagree = 3 Disagree = 2 Strongly disagree = 1

| No. | Social factors at work  (Organization & Procedures)  *R: Reverse-scored question | Answer for organization | | | | | Remarks |
| --- | --- | --- | --- | --- | --- | --- | --- |
|  |  | 5 | 4 | 3 | 2 | 1 |  |
| 1 | Role and responsibility are ambiguous or conflict to others. *R |  |  |  |  |  | Less role  expectation |
| 2 | Personnel have duty of care for other people’s safety. |  |  |  |  |  |  |
| 3 | There are scenarios to where workers don’t have clear guidelines on the tasks they are expected to do (and not do) |  |  |  |  |  |  |
| 4 | There are uncertainty about (or frequent changes to) tasks and work standards. *R |  |  |  |  |  |  |
| 5 | There are too much to do within a certain time or with a set number of workers. |  |  |  |  |  | Less  job demands |
| 6 | There aren’t conflicting demands and deadline pressures for robot-related tasks. *R |  |  |  |  |  |  |
| 7 | There are requirements for excessive periods of alertness and concentration, not with highly repetitive tasks. |  |  |  |  |  |  |
| 8 | I am working with aggressive or distressed people ocassionally. |  |  |  |  |  |  |
| 9 | There is lack of practical support provided to assist workers during transition periods. *R |  |  |  |  |  | Change management |
| 10 | There aren’t prolonged restructuring for robot-related organization and recruitment. |  |  |  |  |  |  |
| 11 | Consultation and communication about workplace changes is lacking; poor quality or untimely. *R |  |  |  |  |  |  |
| 12 | There are personnel working far from home, working in isolated places. *R |  |  |  |  |  |  |
| 13 | There often are personnel working overload or working overtime. |  |  |  |  |  | Less  workload |
| 14 | There are high levels of time pressures for robot-related work hours and deadlines. |  |  |  |  |  |  |
| 15 | There are personnel being continually subject to deadlines of robot-realted tasks. |  |  |  |  |  |  |
| 16 | I have a control of work pace for robot-related tasks. *R |  |  |  |  |  |  |
| 17 | There is lack of variety of work for robot-related tasks. |  |  |  |  |  | Flexible  work schedule |
| 18 | There are flexible work schedules for robot-related tasks. *R |  |  |  |  |  |  |
| 19 | There are unpredictable work hours and overtime ocassionally. |  |  |  |  |  |  |
| 20 | There are fragmented work or works that are not meaningful. |  |  |  |  |  |  |

(6) Collaboration and Communication checklist

* Strongly agree = 5 Agree = 4 Neither agree nor disagree = 3 Disagree = 2 Strongly disagree = 1

| No. | Social factors at work,  (Collaboration & Communication)  *R: Reverse-scored question | Answer for collaboration | | | | | Remarks |
| --- | --- | --- | --- | --- | --- | --- | --- |
|  |  | 5 | 4 | 3 | 2 | 1 |  |
| 1 | Communication between managers and workers are poor in my company or team *R |  |  |  |  |  | Interpersonal  relationship |
| 2 | There are not poor relationships between managers, supervisors, coworkers, and clients. |  |  |  |  |  |  |
| 3 | There are being watched interpersonal conflict or trouble in my company. *R |  |  |  |  |  |  |
| 4 | There are almost no harassment, bullying, third party violence in my company. |  |  |  |  |  |  |
| 5 | There are not lack of sharing and notifying clear vision, objectives, and management information of my company. |  |  |  |  |  | Leadership |
| 6 | Management style of my company is unsuited to the nature of the work and its demand such as suggestion and complaint. *R |  |  |  |  |  |  |
| 7 | There are not adequate communication and support to reflect employees’ needs of my company. *R |  |  |  |  |  |  |
| 8 | There are not inconsistent and unclear decision-making practices in my company. |  |  |  |  |  |  |
| 9 | There are poor communication of robot-related tasks between managers and workers . *R |  |  |  |  |  | Organization  culture |
| 10 | There are low levels of support for problem-solving and personal development. *R |  |  |  |  |  |  |
| 11 | There are sufficient definition of, or agreement on organizational objectives and climates. |  |  |  |  |  |  |
| 12 | There are consistent and timely application of policies and procedures, unfair decision-making. |  |  |  |  |  |  |
| 13 | There are balance between workers' effort and formal/informal recognition and reward. |  |  |  |  |  | Recognition,  Reward, Career |
| 14 | There are lack of appropriate acknowledgement and appreciation of workers' efforts in a fair and timely manner. *R |  |  |  |  |  |  |
| 15 | There are career stagnation and uncertainty, under-promotion or over-promotion, lack of opportunity for skill development. *R |  |  |  |  |  |  |
| 16 | There are adequate support from supervisors and sufficient self-improvement services and training for better performance. |  |  |  |  |  |  |
| 17 | There are lack of positive performance feedback and evaluation processes or lack of support to faciliate performance. *R |  |  |  |  |  | Supervision |
| 18 | Therer are job-related encouragement, acknowledgement, and fairness. |  |  |  |  |  |  |
| 19 | There are sufficient communication and feedback channels in my company. |  |  |  |  |  |  |
| 20 | There are lack of shared organizational vision and objectives. *R |  |  |  |  |  |  |
